# Supplementary material for: Cloud BioLinux: pre-configured and on-demand bioinformatics computing for the genomics community
Source: BMC Bioinformatics. 2012 Mar 19;13:42. doi: 10.1186/1471-2105-13-42 (PMC3372431; doi:10.1186/1471-2105-13-42)
Supplement: Additional file 1 — Supplementary 1 Cloud BioLinux software documentation in the form of a mini, self-contained website. Users need to download and uncompress the .zip file, and open through a web browser the "index.html" file available on the main directory. (ZIP 1823 kb). [file 1471-2105-13-42-S1.ZIP › Cloud-BioLinux-Package-Documentation/docs/lav2ps.html]

Bio-Linux Software Documentation Pages

Back to search form

## lav2ps

|  |  |
| --- | --- |
| Name | lav2ps |
| Description | **lav2ps** is part of the *Fasta3* package of software.  **lav2ps** takes lav format output from lalign and generates a graphical rendition of the results in postscript.   To do this, use the -m 11 flag with **lalign**. This generates lav format output. Pipe the lalign results directly to **lav2ps**:  `lalign -m11 inputSeq1.fasta inputSeq2.fasta \| lav2ps > lalignResult.ps` |
| Homepage | http://www.people.virginia.edu/~wrp/pearson.html |
| Remote Documentation |  |
